# Artificial Muscles: Electrostatic Actuation and Design Tradeoffs

**DOI:** 10.3390/biomimetics11060399

**Published:** 2026-06-05

**Authors:** Gabriel X. Colborn, Justin Pilgrim, Ka Ho, Pragya Natarajan, Arnia Goode, Jeffrey K. Catterlin, Michael Krause, Terak Hornik, Emil P. Kartalov

**Affiliations:** 1 Physics Department, Naval Postgraduate School, 1 University Circle, Monterey, CA 93943, USA; 2 MOVES Institute, Naval Postgraduate School, 1 University Circle, Monterey, CA 93943, USA

**Keywords:** actuators, actuation, linear actuators, electrostatic actuators, muscles, artificial muscles, dielectric elastomer actuators (DEAs), electroactive polymers (EAPs), soft robotics, electrohydraulic, HASEL actuators, ferroelectric polymers, liquid crystal elastomers, microactuators, smart materials, biomimetic actuation, microfabrication, 3D printing, contraction, stress, strain, soft robotics

## Abstract

Artificial muscles are an emerging class of actuators designed to mimic the compliant, efficient, and versatile behavior of biological muscles for fields including the following: soft robotics, prosthetics, wearable enhancements, haptic interfaces, and biomedical devices. These systems encompass various actuation mechanisms, including pneumatic, hydraulic, thermal, ionic, electrochemical, and electrostatic. Each with distinct tradeoffs in voltage, strain, output force, bandwidth, efficiency, and manufacturability. Among them, electrostatic actuators have attracted increased attention due to their fast response times, high energy densities, strong compatibility with soft materials, and scalability from microscale devices to large-area and stacked actuators. However, challenges such as dielectric breakdown, material fatigue, and fabrication complexity continue to limit widespread deployment. This review presents a structured classification of various artificial muscle technologies and an in-depth examination of electrostatic actuators including dielectric elastomers, electrostrictive and ferroelectric polymers, liquid crystal elastomers, electrostatic film motors, stacked architectures, and microscale/milliscale devices. In this review the operating principles, materials, architectures, performance characteristics, and failure modes of electrostatic actuators will be discussed. Additionally, a comparison will highlight tradeoffs across actuator families based on metrics such as voltage, force, strain, bandwidth, and manufacturability. Lastly, we outline future research directions in materials, physics-informed modeling, system integration, and scalable fabrication necessary to advance electrostatic artificial muscles toward practical, real-world deployment.

## 1. Introduction

Artificial muscles are devices that mimic some of the functional behavior of biological muscles, enabling actuation modes such as contraction [1,2,3], elongation [2,3], expansion [3,4], rotation [5,6], and bending [6,7] in response to inputs or stimuli. Common driving stimuli include thermal [6,8], pneumatic [3,7], hydraulic [9,10], electrical [11,12], ionic [1,13], and electrochemical [14,15]. Thermal stimuli can be delivered through Joule heating, convective heating, or photothermal excitation to trigger phase transitions or anisotropic expansion. Pneumatic stimuli uses fluid pressure to inflate elastomer bladders, or use vacuum-driven buckling to generate contraction. Hydraulic stimuli redistribute liquid dielectrics to amplify electrostatic forces or to pressurize tubes. Electrical stimuli involve voltages, currents, and/or electric fields to generate deformation. Ionic stimuli, including electroactive polymers (EAPs), utilize the migration of ions through an electrolyte by the movement of hydrated cations to cathodes to cause localized swelling, bending, and shrinking. Electrochemical stimuli involve chemo-mechanical or reduction-oxidation (redox) reactions triggered by comparatively low driving voltages or environmental pH and humidity variations to induce volumetric changes.

Ongoing research across a breadth of fields seek to integrate artificial muscles into soft robotics [16,17], prosthetics [2,18,19,20,21], wearables systems [18,22], biomedical devices [16,18], and haptic interfaces [9,23], where compliance (flexibility that allows for fitting to a variety of shapes) and smooth continuous biomimetic motion are essential. Compared to traditional rigid motors and transmissions, artificial muscles can offer high power-to-weight ratios [3], inherent compliance [24], and improved safety [17,25] in human environments. Although safety of these new electrostatic actuation technologies is not within the scope of this paper, we will not omit it. Multiple articles have already addressed potential safety concerns, such as high-voltage requirements, robotic-human interaction, water-electronic interference, and operational safety through compliant structures [3,26,27,28]. In an article by S Pourazadi et al., the safety criteria for consumer electronics, particularly for dielectric elastomer actuators (DEAs), based on Underwriters Laboratories (UL) and International Electrotechnical Commission (IEC) standards [29] were listed as an inverse relationship between current strength and time of exposure for a lethal shock.

The growing demand for compact [30], lightweight [23,30], and highly pliable [23,30,31] actuation systems has intensified the need for alternatives. These alternatives would need to be unlike the traditional electromagnetic or hydraulic actuators that tend to be rigid, bulky, inefficient, and/or impractical. Applications such as wearable assistive devices [18,23,32], minimally invasive surgical tools [12], soft grippers (for delicate manipulation) [27,33,34], and tactile haptic interfaces [35,36,37] increasingly require actuators that can safely and organically deform with the human body, interact with fragile objects, or handle high-precision tasks, which are areas wherein traditional rigid motors struggle. As soft robotics and human-machine interfaces continue to grow, artificial muscles have emerged as a critical technology for achieving natural [1,18], biomimetic motion [1,18] in compact and mechanically flexible form factors [12,38].

Artificial muscle technologies are often classified according to their driving actuation mechanisms. Fluidic artificial muscles encompass vacuum-driven [4,26], hydraulic [26], microfluidic [39,40], braided [3], and fabric-based actuators (such as McKibben muscles [3] that employ bladders to be filled with fluid for actuation). Ionic and electrochemical actuators include ionic polymer-metal composites [18,35], conductive polymers [18,35], ionic gels [18,35], ionic EAPs [18], and ionically driven hydrogels [28], which rely on ion transport and electrochemical reactions to generate movement. Thermally driven artificial muscles including shape memory alloys [1,5] and polymers [8], utilize temperature-induced phase transitions to produce actuation [15]. In contrast, electrostatic actuators have a core difference in the way that they generate mechanical deformation through electric field-induced stress in dielectric materials. This also includes architectures such as dielectric elastomer actuators [35,38], ferroelectric [18,35] and electrostrictive polymers [18,41], liquid crystal elastomers [25,38], electrostatic film actuators [12,30], stacked multilayer electrostatic actuators [42,43], and artificial muscles based on fluidic microcapacitors [40,44].

Among these technologies, electrostatic actuators have attracted increasing attention due to their favorable characteristics. Electrostatic actuation mechanisms can enable fast response times [34,37,45], high energy densities and efficiencies [27,28,41,46], and scalability from microscale devices to large-area and stacked actuators [12,43,47], leveraging innovative wiring schemes such as the double-helix ‘weave’ to build scalable microcapacitor arrays [48] and later embodyments inclduing ‘serpinitnes’ and ‘Potatio spirals’ [40]. Additionally, many electrostatic actuators have relatively simple architectures and exhibit strong compatibility with soft, elastomeric materials, making them well suited for applications in soft robotics [9,49], wearable technologies [11,49], and biomedical systems [12,49]. Despite these advantages, challenges such as dielectric breakdown [27,31,34], material fatigue [22], and manufacturing scalability [11,32,35,50] remain active areas of research.

The increasing demand for actuators that can safely and organically deform, either along with or in addition to, the human body has shifted research into muscle-mimetic technologies over traditional rigid actuators. As the field expands to encompass various pneumatic, thermal, and electrostatic mechanisms, the rapid pace of innovation requires systematic, data-driven evaluation of current literature to assess its progress and identify gaps in the research. Bibliometric data highlights this shift with approximately 75% of all artificial muscle research having been published within the last decade (2016–2025), demonstrating the field’s transformation into a massive multidisciplinary effort spanning throughout chemistry [18], material science [51], and robotics [32,33]. By analyzing the publication metadata and keyword co-occurrence, specific technological families, such as dielectric elastomers, liquid crystal elastomers, and hydrogels, can be identified as primary research areas. Likewise by evaluating current research trends, performance records of individual technologies can be highlighted while exposing gaps in the research field.

Given the rapid growth of artificial muscle research and its expanding applications, this review will present a comparative framework that evaluates artificial muscle technologies based on performance tradeoffs and application-driven design criteria while also linking material-level performance metrics with system-level integration and manufacturing constraints. Section 2 will discuss the historical trends and meta-analysis of artificial muscles. Section 3 will briefly discuss potential modern applications of artificial muscles to show why there is a increase of attention to this field. Section 4 provides concise classifications of non-electrostatic artificial muscle actuation mechanisms and metrics, enabling a comparison with electrostatic approaches. Section 5 offers an in-depth review of electrostatic actuator technologies, covering fundamental operating principles, material systems, actuator architectures, associated challenges, and comparing metrics and performance of each type of electrostatic actuator technology. Section 6 examines the impact that advancements in 3D fabrication have on artificial muscles, along with manufacturing considerations and application opportunities. Finally, Section 7 synthesizes key insights, provides the authors’ perspectives, and outlines future directions for electrostatic artificial muscle research over the next decade, with emphasis on overcoming current system-level and manufacturing limitations.

## 2. History, Trends, and Meta-Analysis

The field of artificial muscles is relatively young, starting with Joseph Laws’ McKibben actuators in the 1950s [3]. Then with the later emergence of other categories such as electroactive polymer artificial muscles (EPAMs) in the early 1990s [3,52]. The use of scholarly research papers and patents as part of a bibliometric analysis, first introduced by Pritchard (1969) [53], can be an effective tool to discern the current situation and analyze trends in scientific research in artificial muscles [51].

A query formula, “artificial muscle” or “soft actuator” or “biomimetic actuator” or “compliant actuator”, was devised to retrieve research data from the Web of Science (WoS). Within the WoS, Science Citation Index-Expanded (SCI-E) was selected as a data source due to its status as a leading and trusted citation database covering approximately 9500 scientific journals and more than 70 million records [54]. While this query formula based approach has limitations, this bibliometric method paints a clear picture of the current standing and direction of artificial muscles. The English-language-only query was conducted on 31 December 2025 and the retrieval field was limited to “topic” to search within titles, abstracts, and author keywords. 8004 total documents were collected from SCI-E ranging from the earliest publication in 1961. These documents contained 7185 research papers, 672 review papers, 205 conference proceeding papers, and other published media (e.g., early access articles, editorial material, meeting abstracts, etc.). Metadata from the results of this bibliometric method were then extracted and used for further analysis.

**Figure 1:**
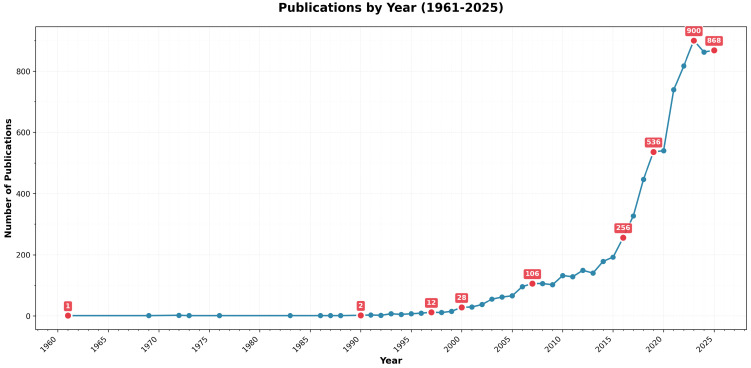
Publications on artificial muscle research over time in the Web of Science database.

The earliest research literature on artificial muscles was published in 1961 within SCI-E and describes the technology of McKibben pneumatic artificial muscles [55]. Figure 1 displays the research trends of the field of artificial muscles through the lens of research publications.

In the decades following 1961, publications on artificial muscles remained low until 1997 where the number of publications surpassed 10. At the turn of the century, a slow, upward trend in artificial muscle research could be seen with 28 publications in 2000 until it broke past 100 publications 106 in 2007. As informational and computing technologies grew, so did research in artificial muscles. The annual publications number more than doubled from 2007 to 2016, and continued to increase for the next decade. From 2016 onwards, annual publications consistently surpassed 256, reaching a record high of 900 in 2023 and a total of 868 in 2025. Artificial muscles has become a significant, multi-disciplinary research effort in the past decade as 75% of all research thus far can be attributed to publications from 2016 to 2025, spanning fields such as material science, engineering, chemistry, nanoscience, robotics, and physics.

**Figure 2:**
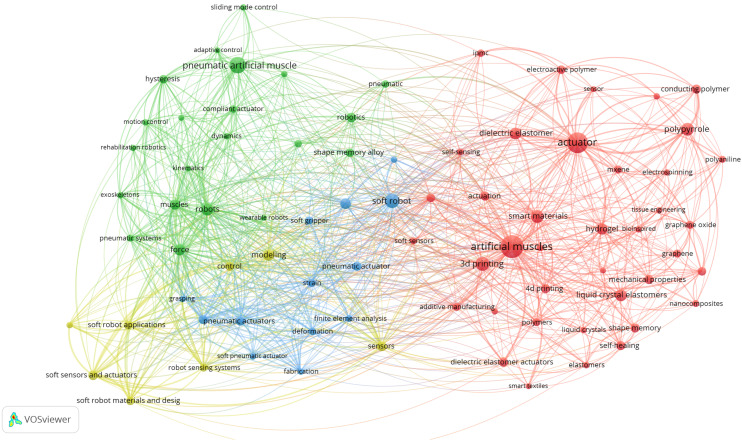
Clustering of research material keywords via a co-occurrence analysis on VOSviewer visualization software.

Keyword co-occurrence analysis was performed on the collected SCI-E research media using a bibliometric network tool, VOSviewer. The results of the clustering based on keyword link strength can be seen in Figure 2. The larger nodes represent increased frequency of papers with those keywords. Closer distances and thicker lines represent increased frequency in the keywords appearing together, suggesting a relationship between those keywords. Lastly, the colored clusters are the 4 largest themes or clusters of ideas, which can be summarized as non-pneumatic artificial muscles (Red), pneumatic artificial muscles (Green), controlling mechanical movements (Yellow), and soft robotic applications (Blue). From a list of the 100 largest-occurring keywords of all time, the following technology categories were identified as hotspots (in order of occurrence): pneumatic actuators, liquid crystal elastomers, dielectric elastomers, hydrogels, electroactive polymer, and carbon nanotubes.

Within the past decade from 2015 to 2025, the list does not change greatly: pneumatic actuators, liquid crystal elastomers, hydrogels, dielectric elastomer actuators, hydraulic/pneumatic actuators, and shape memory alloys. Significant fields, as indicated through recent research, appear to include pneumatic artificial muscles (PAM), dielectric electroactive polymers (EAPs) (i.e., liquid crystal elastomers, dielectric elastomers), and ionic EAPs (i.e., hydrogel-based actuators). The continued relevancy of pneumatic and electrostatic actuators since the start of artificial muscles research highlights their prominence within the field.

To achieve high-performance artificial muscle systems, the literature has largely split into two research directions: (1) pushing performance in individual metrics, and (2) optimizing system-level trade-offs for real-world deployment. Many papers can be classified as exploratory research aimed at charting the limits of artificial muscle technology with examples like the electro–stiffened ribbon actuator (ESRA) [31] and the bilateral actuator design for DEAs [56]. These research efforts provide benchmarks across electrostatic and non-electrostatic actuator families, but are often impractical due to voltage, lifetime, and manufacturing constraints. Some projects, like hydraulically amplified low-voltage electrostatic (HALVE) actuators used in untethered land and sea robotics [27], conduct a hybrid approach that reveals new regions of performance space (reduced driving voltages) while optimizing size, weight, power, and manufacturability for demonstrable, deployable systems. These papers shy away from exploratory material science-based research to additionally consider power electronics, packaging, and reliability for real-world adoption in soft robotics and wearable technologies.

Extended searches into the SCI-E index with the original query formula and additional keywords were performed to further characterize the gap in system integration research from the start of 2015 to the end of 2025. System integration is defined as, but not limited, to any of the following: feedback control, power, electronics, embedded system, closed-loop control, self-sensing capabilities, real-time control, co-designed mechanism, compactness, modularity, and packaging for environmental protection. Search results were logged in Table 1 for several classifications of papers within the engineering design process.

**Table 1:** Extended Database Queries to Characterize Technological Readiness.

Target Paper Type	Additional Keywords	Search Results
Baseline	none ^1^	6507
Manufacturing-Focused	3D printing, additive manufacturing, fabrication, manufacturing	2594
Application-Driven	wearable robotics, exoskeleton, prosthetics, rehabilitation, assistive devices, haptics	778
System Integration	system integration, feedback control, power electronics, embedded system, closed-loop control, sensing and actuation, real-time control	222

^1^ Note. The original query of [“artificial muscle” or “soft actuator” or “biomimetic actuator” or “compliant actuator”] was combined with [additional keywords] to find manufacturing-, application-, and system-based research material in SCI-E from 1 January 2015 to 31 December 2025. The additional keywords make up a set of logical disjunctions.

Approximately 40% of research material from 2015 to 2025 directly references manufacturing and signals growing efforts to produce repeatable and scalable artificial muscle mechanisms. However, only about 12% of the total publications are involved in application-driven work with real-world uses in medical or consumer technology fields. Alarmingly, only ∼3% (222 articles) consider system-level principles necessary for integrated muscle technology to be deployed outside of the lab towards mass adoption.

While both exploratory and optimization-based approaches are essential, literature involving the system-level development of artificial muscles in the last decade is severely lacking (See Table 1). One way to achieve widespread artificial muscle adoption, is to dedicate a larger research focus on functionally complete systems to build off of the dozens of exploratory papers in shape memory alloy (SMA) actuator, hydraulically amplified self-healing electrostatic (HASEL) actuator, and DEA designs [5,34,47]. The successful adoption of artificial muscles into real-world soft and wearable robotics will be determined less by record-setting metrics in energy density, stress, strain, etc. and more by their ability to operate at biologically relevant bandwidths while balancing efficiency, durability, and manufacturability.

The need for actuators that can overcome the inherent limitations of traditional rigid motors, such as high weight, low force-to-weight ratios, lack of physical compliance, and mechanical noise, is the driving reason for the rapid growth of artificial muscle research [16]. As research shifts from material exploration into system-level integration, artificial muscles are proving to be transformative and versatile across a wide range of modern applications where organic deformation or human centered interaction are paramount. These domains include marine and underwater systems, where biomimetic designs enable quiet and stealthy acoustical propulsion; aerospace and aerodynamic morphing; musculoskeletal locomotion; and robotics that mimic the agility of natural organisms and through antagonistic muscle architectures [2]. Furthermore, the scalability of these technologies provides great opportunities in support of industrial automation, defense, and heavy lifting, by allowing lightweight power augmentations and high force-to-weight ratios that rival traditional hydraulic machinery [42,44].

## 3. Modern Applications

Potential opportunities in the future of artificial muscles lie primarily in soft robotics, haptics, wearables, prosthetics, exoskeletons, biomedical devices, and bio-inspired systems. These domains prioritize lightweight and compact form factors; quiet, smooth, and compliant motion; low power consumption and/or power efficiency; and safe operation near humans [33,37,57,58]. These are also particularly applicable to high-stakes defense applications, as biomimetic artificial muscles have the potential to provide stealthy undersea propulsion for ROVs (remotely operated vehicles) and lightweight power augmentation for soldier exoskeletons in environments where traditional motors are too noisy, bulky, or fail to mimic human skeletal kinematics [59,60].

Rather than seeking a universal actuator, future research should adopt an application-driven approach by co-designing artificial muscles with mechanical structures, implementable control strategies, and appropriate power electronics. Hybrid systems that combine multiple actuation mechanisms and fabrication methods may offer a practical path toward overcoming the individual limitations of each actuator type. The large successes of artificial muscles in real-world applications can be seen in research that adopts this multi-modal approach such as HEXEL modules for field robotics and HASEL actuators for untethered, biomimetic robotics [27,45]. Another example of an effective hybrid system is the Peano-Hasel actuator “that combine the strengths of fluidic actuators and electrostatic actuators, while addressing many of their issues” [61]. Designing around key performance requirements like high power density in biomimetic muscles, compliance in soft grippers, tunable stiffness for assistive wearables, fast switching for haptic devices, precision in surgical tools, and modularity in reconfigurable robots will result in the largest success towards adoptable artificial muscles.

### 3.1. Marine and Underwater Systems

Artificial muscles provide a feasible pathway for acoustically quiet and efficient underwater propulsion [44]. More specifically, Dielectric Elastomer Actuators (DEAs) are well suited for this application, with high frequency models enabling swimming robots that can achieve speeds exceeding 5 cm/s in extreme deep-sea conditions [50,62]. The biomimetic designs of these DEAs also allow for minimized acoustic signatures and enhanced stealth for unmanned underwater vehicles (UUVs) [44], where inspiration can be drawn from the concentric contraction of a squid’s mantle to expel water for reactive propulsion [60] or the slow, large-displacement fin motions of pelagic fish, both of which can accomplish these tasks.

Additionally, the feasibility of having integrated underwater locomotion in untethered robotic fish is further enabled through low-voltage electrohydraulic systems, such as HALVE, making these robots functionally waterproof [27]. Similar research has led to the development of biomimetic jellyfish robots using ionic polymer-metal composite (IPMC) actuators allowing for silent and efficient operation [60]. These actuators are preferred over traditional hydraulic systems in gas and oil production facilities in the Arctic region for the repair and maintenance of subsea infrastructure [60].

### 3.2. Aerospace and Aerodynamic Morphing

Electrostatic actuators can find many applications in aerospace, as their high bandwidths and energy densities can be put to good use in agile micro-air vehicles (MAVs), aerodynamic surfaces, and deployable aerospace structures [26]. Such cases are already being reported with dielectric elastomers being used to power insect-scale flight with actuation frequencies of up to 400 Hz [50]. Furthermore, integration of active polymers into flexible airfoils enables seamless shape-changing and ‘morphing’ wings that are capable of adjusting their hydrodynamic and aerodynamic profiles opting over traditional rigid hinges that have inherent complexity and can weigh down the system [5,16]. This can also be achieved through the use of additive manufacturing by embedding high-bandwidth electrostatic actuators with 3D printed lattices [63]. Additionally, 3D printing of sensors and actuators is a priority in satellites for exploration and security, where weight and power efficiency are key design considerations [59]. Although not electrostatic actuators, similar concepts are being employed in shape-memory alloys and pneumatic muscles due to their high-force characteristics in the trailing edge flaps of helicopters to improve stability [3].

### 3.3. Musculoskeletal Locomotion and Robotics

Artificial muscles have also enabled biological levels of agility and adaptability through their integration into antagonistic musculoskeletal structures. By combining electrostatic zipping with hydraulic pressure, jumping robots with spider-inspired electrohydraulic joints can achieve jump heights ten times their own height [9]. Other antagonistic joints can be created by pairing HASEL actuators with electrostatic clutches to achieve full ranges of motion without displacement losses from tendon slack [2,64]. On a larger scale, soft actuators that utilize auxetic and origami bellows to create a robot leg, on the human scale, demonstrate the ability to kick a ball [46].

### 3.4. Industrial Automation, Defense, and Heavy Lifting

The scalability of electrostatic and polymer technologies enables output force production to rival pneumatic and hydraulic machinery, allowing them to find strong uses in industrial applications or general heavy-duty applications. Additionally, kilonewton-scale forces and high force-to-weight ratios of multilayer electrostatic film motors enable them to lift multi-kilogram loads with precise and hysteresis free control [30]. Large-scale stacked actuators (LSEAs) also provide contraction ratios and forces equivalent to mammalian skeletal muscles that can be used in power-assist suits, which can also be applied in industrial settings [42].

Liquid crystal elastomer (LCE) fiber bundles also show potential in high-load industrial applications, as they can achieve loads up to 2500 times their own mass [22]. As a result, biomimetic artificial muscles are highly suited for individual physical therapy assistive devices [65,66]. The high load-to-mass ratio is also applicable in instances of load carriage in defense applications, as it enables movement of heavy equipment over difficult terrain [59]. Additionally, soft micro-grippers designed with additive manufacturing methods can be used to manipulate heavy loads without damaging its surroundings, this is especially useful in subsea applications or other sensitive environments [63].

## 4. Non-Electrostatic Actuation

Artificial muscle methodologies are distinguished by their materials and their modes of enabling actuation responses such as contraction, elongation, expansion, rotation, and bending, all in response to external stimuli [1]. These driving inputs include electrical fields, thermal changes, fluid pressure, and chemical triggers [16]. Besides electrostatic actuators, these include ionic and electrochemical, pneumatic, and thermally driven actuators [3,35].

To evaluate these technologies, the field generally uses several standardized metrics, which are defined in Table 2 [1,3,10,35].

**Table 2:** Standardized performance metrics used to compare artificial muscle actuator technologies.

Metric	Symbol	Units	Definition
**Output Strain**	ϵ	[-]	Relative change in length after actuation, measured as a dimensionless percentage change from its original length, measures movement range.
**Output Stress**	σ	[$\frac{\mathrm{Force}}{{\mathrm{Length}}^{2}}$]	Force generated per cross-sectional unit area. Measures the ability to apply load to the external world.
**Stress-Strain Relationship**	κ	[$\frac{\mathrm{Force}}{{\mathrm{Length}}^{2}}$]	Also represents stroke/load ratio; describes how actuator force or stress changes with deformation, stiffness, and operating condition. Important for assessing wether an actuator has optimized stress or strain at the expense of the other.
**Force-to-Weight Ratio**	*FWR*	[$\frac{\mathrm{Force}}{\left(\mathrm{mass})(\mathrm{gravity}\right)}$]	Also known as Strength-to-Weight; output force normalized by actuator weight. Allows better comparison of smaller actuators and feasibility for size limited applications.
**Power Density**	$P_{d}$	[$\frac{W_{\mathrm{output}}}{{\mathrm{Length}}^{3}}$]	The mechanical work or output normalized to the volume of the actuator, allows benchmarking performance across actuators of different sizes.
**Efficiency**	η	[-]	The ratio between mechanical work output the system provides to energy input the system is provided.
**Bandwidth**	BW	[Hz (range)]	The range of possible actuation frequencies during operation. Also related to the dynamic response time.

Although these metrics are the most reported across the field, they are not intended to be all-inclusive and may need to be expanded on in future work. In fact, success metrics differ according to the goals of different research works. Stress [σ], strain [ϵ] (see Table 2), cycle life, and the elastic modulus [E] may be highly prioritized to mimic natural muscular properties in biomimetic works [12]; electrochemical actuator devices may focus on ion mobility or diffusion [18]; and soft actuator applications may prioritize output strain [ϵ] over output stress [σ] (see Table 2) [56]. The scalability limits for the future of each actuator category are also investigated with extra attention to the physical mechanisms that produce critical design tradeoffs rather than empirical benchmarks alone. In addition, several application domains are matched to each actuator family as some artificial muscles straddle the technology demonstration and subsystem development phases of technological readiness.

At the end of this section, Table 3 was compiled for a comparison summary of the various non-electrostatic actuators.

### 4.1. Ionic and Electrochemical Actuators

Ionic electroactive polymers (EAP) rely on the physical migration of ions through an electrolyte as opposed to static charge accumulation [15]. Some examples are ionic polymeric gels, conductive polymers, and ionic polymer-metal composites (IPMCs).

IPMCs consist of an ion-exchange membrane coated in noble metals, generally an electrolyte, between two electrodes. The deformation of the actuator depends on the movement of hydrated cations toward a cathode [1,16,22]. This diffusion or migration of ions through an ionic liquid electrolyte powered by an applied voltage or electric field causes deformation via localized swelling, shrinking, and/or bending [18,35].

Ionic polymeric gels consist of polymer network chains and electrolyte solutions that are activated through a chemo-mechanical reaction caused by the applied voltage [18,35]. The voltage will move hydrogen ions in or out of the gel and this motion will swell or shrink the gel for actuation [18]. The two main types of gels are aqueous hydrogels and the non-aqueous ionic gels [18,35]. These actuators are made of electrically conductive polymers; when oxidation/reduction reactions occur in these polymers and in conjunction with an ion transport, actuation takes place [1,15]. Ionic hydrogels and IPMCs both require low driving voltage and have a fast response, but the maintenance of their surrounding environment causes difficulties [18,22].

Carbon nanotube yarns and films operate via double-layer capacitance in which injection of ions into nanotubes causes expansion [15]. Conductive polymers utilize redox reactions within an electrolyte where volume change is achieved through a combination of electron transfer and ion movement [15]. While these systems have low operating voltages, typically <5 V, their response speed is directly limited by the rate of ion diffusion [16].

#### Ionic and Electrochemical Actuators: Carbon Nanotube Actuators

One type of ionic and electrochemical actuators that have been shown to be highly scalable are carbon nanotube actuators. These actuators are composed of many individual nanotubes that are bundled into films and yarns [28], leading to relatively high potential for scalable devices. They have been shown to maintain most of their performance metrics across a wide range of scales as they have material properties that make them strong and stiff, however those same properties also inhibit their deformation and strain ([ϵ], see Table 2) capabilities [28]. This can be improved through the twisted and coiled configurations at the cost of response time. However, they can be put in series and parallel to improve output strain ([ϵ], see Table 2), and output force respectively [28].

Scaling carbon nanotube actuators can be a challenge due to the difficulty of extracting and its expensive fabrication processes [28]. In general, IPMCs are often used as fin propulsion in robotic fish wherein stacking several layers increases thickness and enhances propulsion [16]. They can also be enhanced through micro/nanoscaled regulation of graphene oxide to enhance response speed [14], as well as optimizing pre-freezing temperatures during fabrication to ensure consistent pore connectivity for ion transport [13]. Overall, IPMCs are very attractive for micro-scale applications such as micro-robots and minimally invasive medical tools as they can scale down physically while only needing low driving voltages (typically <5 V) [35].

### 4.2. Pneumatic Actuators

Pneumatic actuators, generally called pneumatic artificial muscles (PAMs) are soft and flexible linear actuators that consist of an elastomer bladder inside a mesh sleeve of tube fibers. When the fibers are pressurized, the mesh contracts or extends the bladder depending on the configuration of the braided sleeve fibers. PAMs can produce linear contraction like a biological muscle and can be utilized in robotic platforms, medical applications, and more.

PAMs are distinguished by their mesh sleeves and come in braided muscles, pleated muscles, netted muscles, and embedded muscles [3]. Braided muscles use braided netting around the bladder. The most common braided muscle is the McKibben muscle, where the inner tube and braid are connected at both ends. A ‘pleated muscle’ features pleated folds around the bladder that reduce material strain ([ϵ], see Table 2) when inflated. Netted muscles occur when a mesh with large holes with a braid is woven tightly and then placed around the bladder. Embedded muscles are created when the load-carrying threads used in meshes are embedded within the membrane of the muscle.

PAMs prove themselves cost-efficient and durable, but vibration issues can impact their performance by causing non-linear motion in their actuation. Recent innovations include vacuum-driven systems, which use internal skeletons to buckle or fold under negative pressure [4,26,28]. Some prominent commercial leaders and researchers driving the development and integration of these pneumatic technologies include Bridgestone Corp. [3], Festo AG & Co. [3], the Shadow Robot Company [3], and Engineered Arts Limited [3].

#### Pneumatic Actuators: Performance and Dimensional Scalability

Soft pneumatic actuators (SPAs) are generally limited by output stress [σ] and bandwidth [BW] (see Table 2) requirements depending on specific tasks. Current research indicates that scaling the number of smaller actuators into a parallel packed structure compared to a geometrically equivalent single actuator can enhance overall performance [67]. Experiments have been indicating that using a parallel packed actuator grouping of four actuators showed a 23% increase in output force over a volumetrically equivalent single actuator [67]. The researchers also claim further gains of up to 50% are possible; however, they concede that this works well for SPAs that do not expand significantly as opposed to other PAM actuators [67]. This is due to PAM actuators inherently having interference between closely packed actuators that bulge, decreasing the overall efficacy of the parallel architecture [67].

While traditional PAMs have scalability issues, other variations of PAMs have capabilities to scale in both size and configuration. An example is an X-crossing PAM that demonstrates scalability to parallel, asymmetric, and ring-shaped configurations for several applications to increase output force or output strain ([ϵ], see Table 2) [68]. This X-cross configuration is able to scale up or down in size while maintaining strong load carrying capacities.

### 4.3. Thermally Driven Actuators

Thermal actuators utilize temperature-induced phase or volume changes. Two prominent thermally driven actuators are shape memory alloy (SMA), commonly including NiTi-based systems, and shape memory polymer (SMP) [1,5,6].

The enabling mechanism for SMA and SMP actuators is heat causing deformation and actuation, while subsequent cooling results in recovery to the neutral state through the shape memory effect [69]. SMAs in particular exhibit a temperature-driven hysteresis loop as it transforms between a heated austenite phase and cooled martensite phase which enables high-stress contractions [5,6]. SMPs are newly emerging active polymers that can undergo heated and cooled deformation like SMAs, but can also generate bending and folding motions. The contraction produced by SMA and SMP actuators is small, and there is a high amount of stress on the actuator itself. However, the high power generated by these smaller motions make SMA and SMP actuators appealing in some applications. Twisted polymer actuators utilize anisotropic thermal expansion of highly oriented fibers in twisted coils that translate volumetric expansion into high-stroke linear or torsional motion [15,28]. Thermal actuators offer the highest work/power densities [$P_{d}$]; however, their bandwidth [BW] (see Table 2) is severely limited by their rate of cooling for recovery [5,8,44].

#### Thermally Driven Actuators: Performance and Dimensional Scalability

Thermally Driven Actuators include electro-thermo-mechanical actuators used in Micro-electro-mechanical-systems (MEMS) [47], as well as metallic bimetal (bimorph) actuators [1], SMAs, twisted polymer actuators, and LCEs. They achieve scalability through hierarchical organization of fibers and optimization of thermal diffusion paths [1,8,15]. They can also be arranged in parallel, in series, and in twisting and coiling configurations to increase contractile output force or amplify total output strain ([ϵ], see Table 2) while reducing the overall actuator footprint [1,15]. Thermally driven actuators can stack strands of more than 1000 into bundles to allow lift loads of 5000 times their own weight, demonstrating the viability of scaling thermally driven actuators [8]. Another instance of bundling fibers can be seen with the Bidirectional Rotational Antagonistic (BIRAN) SMA actuator, in which force scaling is achieved by bundling SMA wires [5].

One of the greatest limitations of thermally driven actuators is the rate of heat transfer during actuation and relaxation that dictates the maximum frequency of actuation or bandwidth ([BW], see Table 2) [1]. Due to the reliance on heating and cooling for actuation, the time constant for heat diffusion is proportional to the square of the material’s thickness, meaning that as an actuator scales its diameter, its response time increases at the cost of limiting its bandwidth ([BW], see Table 2) [1]. Conversely, if the diameter is decreased, the diffusion distance for heat decreases, leading to faster actuation and higher peak power densities ([$P_{d}$], see Table 2) [1].

To overcome the overheating issues that are normally associated with SMAs, Barakat et al. made two bundles using one continuous SMA wire to maintain high surface-to-volume ratio for convective cooling [5]. Their overall strategy allows them to achieve higher forces and response times. However, it should be noted that they use externally forced air flow for cooling, as their application was aimed at bio-inspired flight [5]. While SMAs provide strong affinity for scaling in both force and strain ([ϵ], see Table 2), they are still severely limited by their energy efficiencies ([η], see Table 2), and thermal management systems. At the moment, they perform best in small scale applications where heating issues can be easily managed.

### 4.4. Conclusion for Non-Electrostatic Actuation

As summarized in Table 3, non-electrostatic actuators often use lower voltages and more manageable input or driving stimuli methods to generate higher stresses (e.g., PAMs or SMAs). However, at the cost of slower response times, lower bandwidth [BW], or reduced energy efficiency [η] (see Table 2) [1,3,5,28]. For example, fluid-driven origami-inspired PAMs demonstrate efficient actuation with 80 kPa negative-pressure inputs to produce peak power-to-weight ratios of over 2000 W/kg, surpassing natural human muscle power density ([$P_{d}$], see Table 2) [26]. However, one major drawback of such a pneumatic and hydraulic system is low energy efficiency: ∼23% for pneumatic and 59% for hydraulic drives [26].

**Table 3:** Comparison of non-electrostatic actuator technologies’ strengths, limitations, and best-fit applications.

Actuator Family	Primary Driving Mechanism	Typical Strengths	Limitations/ Tradeoffs	Best-Fit Applications	Representative Sources
**Ionic & Electrochemical** Including IPMCs, ionic gels, hydrogels, CNT yarns	Ion migration, redox reactions, electrolyte swelling, double-layer capacitance	Low driving voltage, flexible, lightweight, microscalable	Response speed, electrolyte maintenance, output stress [σ], long-term stability	Micro-robots, robotic fish fins, biomedical tools, soft bending devices	[1,13,14,15,16,18,22,28,35]
**Pneumatic Artificial Muscles (PAMs)** Including McKibben, braided, pleated	Pressurized air or vacuum changes bladder/sleeve geometry	Output stress [σ], relatively low cost, durable, mature	Bulky, tethering, nonlinear behavior, vibration, precision, miniaturization	Rehabilitation robotics, exoskeletons, grippers, industrial assistive devices	[3,4,7,17,26,28,67,68]
**Hydraulic & Fluid-Driven** Including origami-inspired fluidic muscles	Fluid pressure or vacuum drives deformation of a flexible shell, skeleton, or folded structure	Very high strain [ϵ] and power density [$P_{d}$], broad material compatibility	Fluidic hardware, tethering, bulky, efficiency [η] varies drastically	Wearables, exoskeletons, deployable structures, soft grippers, high-strain [ϵ] robotic systems	[10,26,28]
**Thermally Driven** Including SMA, SMP, twisted/coiled polymers, photothermal fibers	Joule heating, external heating, anisotropic expansion, or shape-memory recovery	High work density, compact form factor, output stress [σ], useful for small or high-load mechanisms	Hysteresis, thermal inefficiency, fatigue, thermal management, bandwidth [BW]	Small-scale mechanisms, origami robots, morphing structures, compact high-force actuation	[1,5,6,8,15,28,69]

Though the origami-inspired PAM system is simple to build and is safe under negative-pressure operation, it requires bulky external pumps. This results in slow response, limited precision, and the need for tethered operation [26], which all limit practical applicability. SMAs suffer from thermal inefficiency, but also from significant hysteresis due to their phase transformations, which increases difficulty in efficient control [1]. Ionic actuators are constrained by electrochemical properties found in ionic liquid, aqueous, and organic electrolytes, with limited power-to-weight ratios of less than 244 W/kg [1]. Overall, even with the inherent drawbacks of non-electrostatic actuators, they still dominate the landscape of rehabilitation and assistive exoskeletons, despite their bulkiness and mechanical complexities [70].

While non-electrostatic artificial muscles like PAMs can operate at lower voltages and can have better efficiencies [ϵ] they leave parameters like output stress [σ] or force-to-weight ratios un-optimized [FWR] (see Table 2). This introduces the niche that electrostatic arcuation fills.

## 5. Electrostatic Actuation

Electrostatic actuators use electric fields to generate mechanical work by attracting or repelling charged or polarized materials, and can therefore be thought of as ‘electric muscles’ that turn electric potential into motion or force without requiring traditional motors, gears, or pressurized fluids. In practice, electrostatic force generation is typically leveraged either directly, with electric potential and dielectric elastomer actuators, or indirectly, in systems that combined electrostatic advantages with traditional actuators such as electro-hydraulic and HASEL-type devices where electrostatic pressure acts in a confined fluid that then drives a flexible shell. In both cases, using the standardized metrics previously established in Section 4 (Table 2) designers optimize and trade off between required voltage, output stress [σ], achievable output strain [ϵ], bandwidth [BW], fabrication complexity, and robustness to arrive at an actuator that fits a particular application such as soft robots, haptic displays, or biomedical tools.

Across literature, electrostatic actuators are broadly described as capacitive structures in which an electric potential establishes an electric field between pliable or rigid electrodes separated by a dielectric layer [1,39,50], generating a Maxwell stress, an electrically induced pressure, that squeezes the dielectric parallel to the field direction and causes it to expand in-plane [12,35], much like pressing down on a rubber sheet causes it to bulge outward [1,16,35] at a scale dependent on the Poisson ratio of the compressed material. The resulting output stress ([σ], see Table 2) scales with the dielectric permittivity and the square of the electric field, so much of the field focuses on material selection (high-permittivity, high-breakdown-strength polymers, gels, or liquids) and device geometry (narrow electrode gaps, large electrode area, lateral arrays, and multilayer stacks) to maximize useful work output while avoiding dielectric breakdown, electrostatic pull-in, and charge leakage pathways that would otherwise limit reliability [22].

Recent developments in electrostatic actuators have demonstrated how well-suited they may be for artificial muscles when compared to non-electrostatic actuators [1,28,41,50]. Generally, electrostatic actuators boast faster response times, higher strain [ϵ], and higher bandwidth [BW] (see Table 2), which make them suitable for controllable motions that are agile and precise [1,32,43,50]. Furthermore, many electrostatic actuator systems are self-sensing with known mechanical and electrical parameters such as stress, capacitance, and electric potential, which enable closed-loop feedback control that promises stability in real-world situations [2,34,37,49].

A coupled pair of cable-DEAs has been presented to serve as an all-in-one “actuator, stiffness controller, and sensor, enabling closed-loop control of an end effector’s position and compliance” [24]. However, electrostatic actuators typically require very high driving voltages, often in the kilovolt range to generate effective Maxwell stresses, potentially necessitating specialized power electronics and robust insulation [1,35,50]. For example, bilateral DEA have been found to require input voltages as high as 6 kV to achieve effective deformations [56]. Failure modes often involve electrode separation in composite materials, material fatigue under cyclic loading, and “wrinkling or breakdown of the dielectric elastomer” for DEAs in particular [56].

Electrostatic actuators have a promising ability to simultaneously maximize strain [ϵ], stress [σ], speed, bandwidth [BW], and power density [$P_{d}$] (see Table 2). Researchers must balance these prevalent engineering tradeoffs when selecting a technology for a given application [28,32].

Current works highlight two key cross-cutting design themes: (1) dielectric engineering and charge dynamics, in which matching charge relaxation times between solid films and dielectric liquids can suppress interfacial charge buildup, slow force decay under direct current (DC) or low-frequency drive [31,36]. This reduces energy losses by orders of magnitude, enabling nearly constant-force performance from multilayer electrostatic artificial muscles and haptic devices [36]. (2) Architectural amplification, where hierarchical stacking [39,42,43], hinge-guided plate arrays [42], zipping geometries [34,50], and cable or linkage transmissions [24] convert small field-induced thickness changes or shearing deformations into large strokes or torques at the device level. This allows mesoscale robots, grippers, artificial limbs, and microfluidics [40,71] to achieve muscle-like motions even when individual layers deform by only a few percent [12,24].

Electrostatic actuators can be made with various materials but are most often made with electroactive polymers (EAPs). Dielectric EAPs consist of, among others, dielectric elastomers, ferroelectric polymers, electrostrictive graft elastomers, and liquid crystal elastomers [18] which will be further discussed in detail throughout this section and Table 4 will help summarize key comparisons between the various electrostatic actuator types.

### 5.1. Dielectric Elastomer Actuators (DEAs)

**Figure 3:**
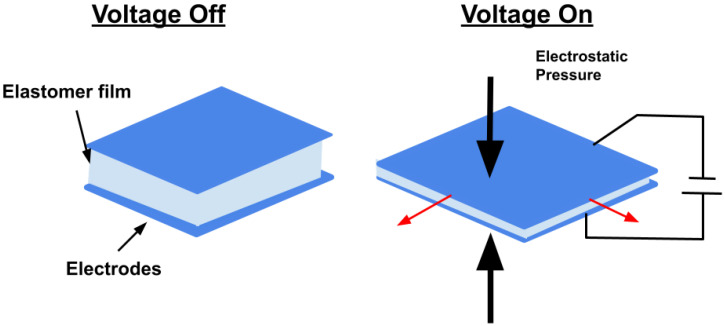
Working principle of a Dielectric Elastomer Actuator (DEA) from reference [18].

Dielectric elastomer actuators (DEAs) consist of a thin, soft elastomer film (typically acrylics, silicones, or polyurethanes) sandwiched between cooperative electrodes [22,43,50]. When connected to a voltage source, the opposing charges on the electrodes are attracted to each other, producing a Maxwell Stress or an electrostatic force that results in the deformation [1,19,22,44,50,72]. This deformation of the elastomer film through the compression of the dielectric material (Figure 3), mimics muscle movement. These electrode are restrained to enhance actuation strain ([ϵ], see Table 2), increase stability against pull-in, and homogenize the electric field so that the entire film participates in the motion rather than localizing near defects (Figure 3).

Under high voltage, the film thins and expands in-plane (see red arrows in Figure 3) as electrostatic attraction between the electrodes compresses the thickness (see bold black arrows in Figure 3). Stacked or rolled configurations increase work output and enable cable-driven or remote actuation of mechanisms such as grippers, multi-DOF (degree of freedom) linkages, and soft end-effectors, making DEAs a canonical platform in soft robotics and bio-inspired artificial muscles [32]. DEAs differ from IPMCs in that they operate through the accumulation of opposite charges on pliable electrodes that generate the Maxwell stress required for actuation. DEAs have no moving charges while ionic actuators rely on physical migration of ions through an electrolyte via electrodes [16,28].

Recent DEA work emphasizes these two directions: (1) functional integration, where DEA membranes have been used as active walls in microfluidic channels, providing voltage-controlled area change or shear to adjust hydraulic resistance for adaptive filters, peristaltic pumping [39,50], and in situ declogging within lab-on-chip devices, and (2) additive manufacturing and patterned electrodes, where embedded 3D printing of interdigitated electrodes within self-healing polyurethane dielectrics has enabled in-plane contractile DEAs with tunable stiffness, breakdown strengths around a few tens of volts per micrometer, and multi-axial voxelized actuation patterns, without requiring large biaxial restrain [39,49,50].

#### Dielectric Elastomer Actuators: Performance and Dimensional Scalability

DEAs achieve performance scalability through geometric tuning of the active area and the number of active layers, the adoption of rolled or fiber form factors, and hierarchical groups of units into parallel bundles or serial stacks [15,43,47]. DEA outputs are driven by its dimensions and can be configured based on application requirements. The force generated is directly proportional to the active area. Increasing the active area in that configuration results in a linear increase in force as the material stiffness and mass do not interfere with the output [43,47]. Actuation stroke scales proportionally with the number of active layers. In free displacement conditions, additional layers contribute their own deformation while decreasing the overall stiffness of the system [43,47].

To overcome the volumetric limitations of planar films, DEAs are scaled into 3D architectures. Rolled DEAs are fabricated by rolling DEA films in multiple layers for a compact volume, to increase the capacity for higher loads, and to increase cycles [37]. Rolled DEAs are especially useful for translating planar expansion into linear tensile strokes [24,37]. DEAs can be scaled into fibers to allow hierarchical bundling [15] like biological muscles, in parallel configuration to amplify output force, or in series configuration to amplify strain ([ϵ], see Table 2) [15]. As DEAs scale in size and complexity, they run into physical and electrical limitations. Interlayer reliability can be compromised in a 3D printed DEA in which electromechanical instability or total dielectric breakdown occurs [43]. Although scaling the number of layers increases the capacitance and decreases the total resistance, it can reduce the electrical cutoff frequency, effectively limiting the maximum operating frequency [47]. Additionally, because DEAs are made from viscoelastic polymers, they exhibit creep that is more pronounced at larger scales and longer operating times [37].

Despite impressive power and strain ([ϵ], see Table 2), persistent challenges for DEAs include material fatigue under large cyclic strains, and fabrication of uniform thin films with robust, low-resistance malleable electrodes at scale, all of which limit translation into consumer or biomedical products.

### 5.2. Ferroelectric and Electrostrictive Polymer Actuators

Ferroelectric polymers extend the electrostatic paradigm by exploiting field-induced polarization changes beyond simple para-electric behavior, enabling higher effective charge density and sometimes built-in spontaneous polarization for larger output stress ([σ], see Table 2) at lower voltages compared to conventional dielectric elastomers [18,35,73]. Electrostrictive graft elastomers are flexible, macromolecular polymer chains that are grafted crystalline pendant groups. The alignment of these molecules due to polarization is changed by the application of the electric field, creating the actuation response [37]. EPAMs have been systematically analyzed utilizing the following: linear elastic theory, with closed-form expressions for compressive pressure; electric field, which is required for a given load; volumetric strain energy density; design relations that have been applied to spherical joint actuators; and rotary motors, giving engineers relatively direct sizing rules for these devices [18].

A particularly notable development across literature is the use of ferroelectric nematic liquid crystals as the dielectric medium in electrostatic actuators, where spontaneous polarization maintains large internal charge densities even at modest applied voltages, dramatically boosting Maxwell stress for a given field [74,75]. By filling a 3D printed double-helical electrode geometry with a customized ferroelectric nematic fluid, researchers achieved forces roughly three orders of magnitude greater than conventional insulating oils in the same field [25]. This research also showed visible contraction at tens of volts and strains ([ϵ], see Table 2) around 19% at fields on the order of 1 MV/m, making battery-driven electrostatic artificial muscles increasingly plausible [25]. These results underscore how material discovery can directly lower the high driving voltage of electrostatic actuators, but ferroelectric media introduce new issues in thermal stability, synthesis complexity, and long-term reliability that remain relatively unexplored at system-level [25,32].

#### Ferroelectric and Electrostrictive Polymer Actuators: Performance and Dimensional Scalability

Ferroelectric and electrostrictive polymer actuators, such as Polyvinylidene Fluoride (PVDF) and its copolymers, achieve scalability through multilayer stacking, molecular phase optimization, and the usage of high-permittivity relaxor-ferroelectric thin films [18,27,35]. Similarly to other electroactive polymers (EAPs), ferrorelectric systems can scale their force [24,35] and displacement [24] outputs through multilayer stacking using ten to hundreds of alternating layers, allowing engineers to scale these actuators for specific requirements. Piezostacks are also utilized for large strain-stress ([κ], see Table 2) actuation, while other alternatives like piezotubes, can be used to scale down performance for nanomanipulation applications [1]. During fabrication, the performance of electrostrictive and ferroelectric actuators can be tuned at the molecular and structural levels.

For electrostrictive graft elastomers, elongation can be scaled by increasing the ratio of polar grafted groups or by increasing material crystallinity through prolonged annealing [18]. Ferroelectric polymers such as PVDFs require maximizing the crystalline β-phase content via mechanical stretching, high-voltage electrical poling, or nanocomposite engineering fillers such as barium titanate to scale the piezoelectric response [35]. The frequency scalability of electrostrictive copolymers, such as high-energy electron-irradiated (HEEI) polyvinylidene fluoride-trifluoroethylene (PVDF-TrFE), are well suited for high speed applications as they exhibit a flat frequency response of up to 100 kHz [1,35]. A major challenge in scaling most electrostatic systems are the large driving voltages due to “bulky and inefficient driving electronics” [27]; however, work is being done to mitigate these issues. By replacing standard linear dielectrics, such as BoPET, with relaxor-ferroelectric terpolymers, driving voltages can be scaled down by 4.9 to 6.6 times, while still maintaining equal stress-strain ([κ], see Table 2) output [27].

Additionally, these materials enable the creation of HALVE actuators which operate at volt to kilovolt scales, using compact battery power electronics for untethered robots [27]. Similarly to other actuators, advances in 3D printing have enabled the scaling of these complex actuators with higher accuracy and precision (see Section 6). Through the use of direct ink writing (DIW) and fused filament fabrication (FFF) (commonly referred to interchangeably with fused deposition modeling or FDM), customized ferroelectric devices can be manufactured on substrates, allowing for integrated 3D microactuators with sub-100 nm features [23,35].

Additionally, manufacturing of these actuators into electrospun aligned nanofiber mats provides high surface-to-volume ratios and porosities, lowering electrical power consumption, and improves viscoelecastic relaxation speeds, further enabling efficient scaling [18]. Lastly, improvements in self-healing properties by integrating relaxor-ferroelectric layers into electrohydraulic structures allow increased durability at scale, enduring multiple dielectric breakdowns while remaining operational [27].

### 5.3. Liquid Crystal Elastomer Electrostatic Actuators

Liquid crystal elastomers (LCEs) appear within the broader EAP research field as a distinct electronic EAP class that combines rubber-like elasticity with anisotropic, field- or temperature-responsive ordering of mesogenic units, allowing large, directional shape changes when the internal molecular alignment is altered [33,76,77]. While many LCE artificial muscles are thermally or optically driven, electrostatic actuation becomes relevant when LCEs are configured as dielectric elastomers (with director alignment controlling anisotropy of both mechanical stiffness and permittivity) or when they are integrated into composites that respond to electric fields via dielectric or ferroelectric effects, enabling bending, twisting, or contraction under applied voltage in a programmable manner [8,76]. Liquid crystal elastomers are also ferroelectric [18,77], can spontaneously polarize in dielectric material, and are extremely flexible. They also generally need lower applied voltages than other DEAs, but have slower response times [76,77].

Reviews position [35,60] LCE-based EAPs as promising for large, programmable deformations and complex shape morphing. For example, morphing shells or soft robotic skins that can change curvature on demand [63]. However, they also note that practical electrostatic LCE actuators are less mature than classical acrylic or silicone DEAs, with fewer demonstrations of high-force, high-bandwidth ([BW], see Table 2) devices [32,35,70]. The literature emphasizes the need for improved synthesis routes that control crosslinking and mesogen alignment [15,35], better electrode–elastomer interfaces that can sustain large anisotropic strains ([ϵ], see Table 2) without delamination [14,35,78], and scalable alignment processes (e.g.: through mechanical stretching or surface patterning), before LCE electrostatic actuators can be widely deployed in soft robots or biomedical devices [8,35,59,63].

#### Liquid Crystal Elastomer Electrostatic Actuators: Performance and Dimensional Scalability

LCE electrostatic actuators achieve performance scalability through hierarchical fiber bundling, multiscale additive manufacturing, and molecular-level structure design [8,22]. Hierarchical bundling and performance multiplication of LCEs are inherently scalable through stacking of individual fibers to mimic biological skeletal muscles, allowing multiplication of work capacity and force proportional to the number of fibers gathered [8]. Through the use of DIW, meter-scale LCE fibers can be produced, which could be a pathway into macro-scale robotic components [15] as well as bio-inspired spinning techniques which can create micrometer-sized fibers (as small as 2.6 micrometers) that maintain high contractile force and rapid response times [15]. By increasing the laminate thickness of LCE’s, their power-to-weight ratios can be increased significantly without changing the material’s inherent deformation behavior [22].

Scalability in force density is often limited by the weakening of the material during contraction, which is addressed by integrating nanofiller [8]. By incorporating exfoliated graphene fillers, a reversible percolation network can be induced that preferentially assembles along the fiber axis during integration [8]. This structural reconfiguration reinforces the mechanical properties of the fiber specifically in its actuated state. This allows the system to withstand large external loads that normally cause pure LCEs to fail [8]. LCE actuators are able to scale their response by integrating conductive elements and utilizing phase-transition mechanics [18,31,35]. Additionally, ferroelectric LCEs utilize molecular reorientation to produce large strains ([ϵ], see Table 2) at operating voltages 100 times lower than standard DEAs, typically in the 1.5–5 kV range, making them applicable at smaller scales [18,22].

### 5.4. Film Motors and Electrostatic Film Actuators

Electrostatic film motors represent a distinct line of work in which patterned electrode films create traveling or overlapping electric fields that produce linear or rotary motion similar to a motor, but in a fully planar or stacked film architecture without magnets or conventional rotary hardware [37,50]. Multi-layer electrostatic film motor actuators composed of numerous double-sided drive films immersed in dielectric fluid have demonstrated high force-to-weight ratios ([FWR], see Table 2) [31,79]. For example, prototypes with masses of a few hundred grams achieving forces on the order of hundreds of newtons at kilovolt-level drive, and lifting multi-kilogram loads. This greatly exceeds comparable actuators of similar size, highlighting the potential of these ‘film muscles’ for high-performance robotics [30]. However, these systems require extended ripple analysis to account for mutual capacitance between phases and the resulting thrust force variations [50,80].

These film muscles are attractive because they exhibit minimal hysteresis, enabling precise position and force control, and their laminar structure is inherently compatible with microsystem and circuit board fabrication technologies, so that they can be produced with standard manufacturing tools [31,74]. Related work at the mesoscale uses hierarchically stacked electrostatic film actuators with microfabricated electrode arrays to achieve strokes on the order of centimeters and forces of tens of millinewtons from volumes of only a few cubic millimeters. This is able to power earthworm-inspired crawling robots and flexible endoscopic tools that conform to tight, curved spaces [37]. The primary limitations are the need for precise assembly and robust mechanical frames to manage many thin layers, as well as continued reliance on kilovolt-level actuation for peak performance [30,37].

#### Film Motors and Electrostatic Film Actuators: Performance and Dimensional Scalability

Electrostatic film motors and film-based actuators achieve scalability in performance through multilayer stacking, double-sided drive configurations, and integration of flexible printed circuit boards (FPCB) technology [30,50]. The scalability of electrostatic film motors is largely dependent on the multiplication of active surfaces [30]. Through multi-layer film stacking, the thrust force of these motors can be linearly multiplied. A multi-layer prototype can produce (2n−1) times more force than a single layer, where *n* is the number of layers in a film [30]. Through this method, the force-to-weight ratio ([FWR], see Table 2) is significantly increased, achieving ratios nearly five times higher than previous rigid and bulky structures [30]. Another technique used to increase the performance of multi-layer actuators is to design films in which the electrodes are equidistant from both the top and bottom surfaces, allowing the use of both sides of the film for driving, effectively doubling their performance [30].

Film-based actuators often utilize a zipping mechanism, where electrostatic attraction draws two flexible insulated electrodes together [31,50]. The force generated by film actuators can be scaled by using a high-permittivity liquid dielectric droplet at the zipping point to amplify Maxwell stresses [31], allowing devices to maintain high performance using thinner dielectric films and lower operating voltages [10,31].

Electro-ribbon actuators (ERA) can be scaled by constructing complex 3D lattice structures or origami-inspired heterochiral stacks, allowing the transition from contraction to rotation and bending [50]. A critical constraint of these actuators is the trade-off between stroke and load. High-stiffness electrodes enable higher contractile forces but smaller strokes, while lower-stiffness films produce larger displacements, but smaller forces [31]. However, advanced designs such as the electro-stiffened ribbon actuator (ESRA) utilize electroheological fluids, to independently scale the bending stiffness and damping of the films [31], allowing for force output adjustments. Additionally, advances in FPCB and 3D printing allow for more precise mass production of fine electrodes on thin polymer films [30] and the fabrication of integrated film-based actuators with a micrometer scale [23,50], potentially increasing stress ([σ], see Table 2).

### 5.5. Stacked Electrostatic Actuators

Stacked electrostatic architectures are a route to higher forces and longer strokes without excessively reducing gap widths, which would otherwise increase breakdown risk and make fabrication more demanding [39,43,44]. Large-scale stacked-type electrostatic actuators (LSEAs) use arrays of parallel rigid plate electrodes separated by combined solid and fluid dielectrics, with hinge mechanisms and external tensile elements to prevent overextension of the gaps during contraction and to keep the plates aligned, thereby maintaining high stress ([σ], see Table 2) across a long-stroke ‘accordion’ stack [37,42]. Experiments show contraction ratios comparable to mammalian skeletal muscle while retaining high force output. This indicates that LSEAs are promising candidates for life-sized robotic artificial muscles and assistive devices where human-scale motion and load-bearing capability are needed [34,42,47].

#### 5.5.1. Stacked Electrostatic Actuators: Performance and Dimensional Scalability

Stacked Electrostatic Actuators (SEAs) or LSEAs achieve performance scalability through geometric manipulation of active areas and hierarchical stacking of layers [42,43,47]. There are two primary stack level design parameters in SEAs/LSEAs: (1) the active area and (2) the number of active layers [47]. The active area determines the blocking force generated [43,47], which has a linear relationship with the force output in a blocked configuration. Dynamic properties, such as stiffness and mass, do not interfere with the generated force [47]. While the number of layers modulates the actuator displacement and affects the stiffness. Increasing number of layers contributes to decreased stiffness and increased displacement [47].

Together, both of the active area and active layers parameters can control the resonance frequency of the actuator [47]. One way for SEAs/LSEAs to scale force output is by enlarging the electrode area per film or increasing the number of interdigitated electrodes connected by hinge parts [42]. The scalability of these actuators is further enhanced by advancements in additive manufacturing and specialized structural architectures [42,43].

#### 5.5.2. Conducted Simulations of SDEAs, SEAs, and LSEAs

Multiple simulations proving the ideas of stacking electrostatic actuators have been conducted. Coltelli et al. [44] showed that electrostatic actuators stacked at 100 micron scale of membrane and electrode thicknesses have their output stress ([σ], see Table 2) saturate around 9 kPa. The saturation was established by consecutive simulations sweeping parameters to maximize force density of arrays of NxNx10 microcapacitors, where N was varied from 1 to 13. The simulations demonstrated that saturation was achieved at around N = 10. That established that no further increases of array size were necessary to extrapolate the result to arbitrary array size, thereby solving a major computational capacity problem. Furthermore, as force density scales with the inverse square of electrode separation, the results suggested that the optimized system would output 900 kPa at the 10 micron scale when array size is increased, which is enough for many conceivable applications.

Another simulation work [72] reported on optimization process of air gaps introduced within the dielectric to facilitate lateral expansion and non-linearly increase output stress ([σ], see Table 2), helping optimize the metrics for SEAs and LSEAs for more effective use [72]. The work demonstrated that the introduction of air gaps increased the expected output stress [σ] by 15% compared to the same architectures without air gaps. As a result of the output stress [σ] increase and the weight decrease from the air gaps, the final product’s Force-to-Weight ratio ([FWR], see Table 2) increases by a significant margin. This more easily enables applications that require a lower mass, but more importantly increases the output stress ([σ], see Table 2). Additionally, the air gaps allow room for more flexibility and biomimetric movement [72].

Overall, these simulations demonstrate that air gaps provide a pathway to increased stroke, though they require precise optimization of gap geometry and muscle-to-tendon ratios to mitigate performance bottlenecks [72].

### 5.6. Challenges and Limitations Across Electrostatic Actuators

#### 5.6.1. High Driving Voltages vs. Integration

Classic DEAs, film motors, and many stacked actuators typically require inputs at the kilovolt scale to generate useful Maxwell stress [28,39,50]. That complicates power electronics design, and insulation, particularly for wearable or biomedical devices that must operate near or in contact with the human body [1,27]. Some studies explore approaches to lower voltage requirement [25], while others say that high-voltage systems often rely on “bulky and inefficient driving electronics” [27]. Note that high voltages are not difficult to generate. For example, a low-cost electroporator using piezoelectric crystals from stove lighters generated 2 kV [81]. Additionally, electrostatic discharge (ESD) from the human body can reach tens of kilovolts under typical conditions as documented in aerospace and electronics standards [82,83]. These examples show that kilovolt-range voltages are not in principle prohibitive in compact and accessible systems. As a result, reducing the driving voltage of DEAs should be viewed primarily as optimizing metrics related to efficiency ([η], see Table 2), controllability, insulation, and system integration, rather than a strict barrier to implementation.

Low-voltage strategies to help simplify these challenges (ferroelectric fluids, high-permittivity liquid crystals, and matched-dielectric electro-hydraulics) are promising but still early-stage [27,31], and they introduce their own constraints on temperature range, frequency response, and long-term reliability [31,50].

#### 5.6.2. Material Durability and Failure

Large cyclic strains in elastomers, strong fields in thin films (where the films also provide high voltage insulation to form the fields), and fluid–solid interfaces in HASEL-like systems lead to dielectric breakdown, mechanical fatigue, and leakage [27,43,50]. Self-healing liquids and self-clearing breakdown behaviors can mitigate some of these issues but do not yet eliminate lifetime and reliability concerns under realistic loading and environments [24,27,34]. As a result, many reported electrostatic actuators still operate primarily under laboratory conditions. This gap between laboratory demonstrations and realistic operating conditions highlights two concerns: (1) translating them into usable products; this requires systematic studies of degradation mechanisms and improved materials that combine toughness, high permittivity, high breakdown voltage, high relative permeability, and high breakdown strength. (2) The lack of system integration research (Table 1); which implies that there is a gap in research on material durability and lifespan with respect to electrostatic muscles per application; and since the field is still in its infancy, there is minimal current research on the cycle-count and fatigue of electrostatic actuation systems.

However, there are several studies that have presented initial performance metrics for cycle life tailored to specific architectures. HASEL actuators have demonstrated the ability to surpass 1 million cycles lifting loads of 150 g at a strain of 15% [ϵ] with no perceivable performance loss [34]. Similarly, silicone-based DEAs have reportedly reached ranges of hundreds to several million cycles at specific electric field strengths [84,85,86], with the failure criteria defined as material strength, dielectric breakdown, and pull-in [87,88]. However, performance for “...a film element made of VHB 4905/4910...” (a type of acrylic common for DEA testing) [87] varies drastically based on environmental factors; unencapsulated VHB films exposed to contaminants fail after 150 cycles, and with protected films fail after over 500,000 cycles [86,87,88,89]. These initial findings suggest that high cycle counts can be achieved in controlled settings. However, it should be noted that these studies do not apply to the field of electrostatic actuators as a whole, and its reported lifespan, cycle counts, and fatigue testing results are values for each individual prototype, as opposed to the relevant family of actuators or all electrostatic actuators altogether. Although these articles provide quantified values for the durability/lifespan of electrostatic actuators, they should not be thought of as general metrics for future research expectations, as the field is still being developed, refined, and changed. Additionally, these assessments come from prototypes and in lab testing, so they may not be representative of the real world and may not even apply to future iterations of specific developing actuators.

#### 5.6.3. Trade-Offs Between Stress, Strain, Bandwidth, and Scalability

Architectures that achieve long stroke (LSEA and origami-like assemblies) often sacrifice simplicity or bandwidth [BW] because they involve many moving parts and larger masses [26,42], while high-bandwidth ([BW], see Table 2) stacked and microscale devices can have limited stroke or require complex fabrication and packaging [47,50]. Currently, no single electrostatic design matches biological muscle simultaneously in strain [ϵ], stress [σ] efficiency [η] (see Table 2), lifetime, and adaptability. As a result, current systems typically optimize one or two of these metrics for a given use case rather than providing a universal solution.

#### 5.6.4. Modeling, Control, and Hysteresis

While some classes (film motors, carefully designed stacks) offer relatively linear responses that can be captured with simple lumped models [30,47], many dielectric elastomer based actuators exhibit strong nonlinearity, viscoelasticity, and hysteresis [37,50]. This complicates closed-loop control and motivates specialized models such as Prandtl–Ishlinskii operators, Preisach-type models, or coupled electromechanical circuit analogies [11,47]. Developing controller-friendly, low-order models that still capture key behaviors remains an active research area, particularly for applications where precise tracking, fast response, or human–robot interaction is required.

Future progress will depend on coupling advanced materials discovery with multi-scale fabrication and integrated sensing and control, moving electrostatic actuators from promising artificial muscles in controlled experiments toward robust, energy-efficient, and safe components of real-world soft robots, prosthetics, and biomedical devices.

### 5.7. Performance Metrics and Tradeoffs

#### 5.7.1. Electrical Requirements

For electrostatic actuators, large electric fields produce fast and efficient actuation, but then insulation and durability become key challenges especially when used in wearable biomedical applications. DEAs operate at around 2–5 kV [39]. HASEL actuators have input voltages in the 5–10 kV for standard designs. HALVE actuators have demonstrated high performance with voltages as low as ∼1.1 kV [27]. Multi-layer electrostatic film motors operate at 1.3–1.8 kV, which has a 4.7 times larger force-to-weight ratio ([FWR], see Table 2) compared to the previous work in the field of film actuators [30]. Ferroelectric liquid crystal actuators [25] produce visible motion at a mere 18 V and significant contractions (19%) at 200 V. Ferroelectrics represent an exciting opportunity to shift to low-voltage electrostatic actuation. With electrostatic actuators being fundamentally capacitive in load type, total power consumption can remain low despite the high voltage due to minimal current draw. Matching the bulk charge relaxation rates of dielectric materials in electrostatic actuators reduces power loss for a constant-force output by up to 3 orders of magnitude under DC operation [36].

#### 5.7.2. Mechanical Properties

Materials that achieve large strains ([ϵ], see Table 2) often do so at the expense of force output, while high-stress actuators tend to be stiff and less compliant. Hexagonal electrohydraulic modules can achieve up to 49% linear contraction with peak strain ([ϵ], see Table 2) rates of 4618% per second [45]. Meanwhile, HALVE actuators reach peak strain rates of 971% per second matching mammalian skeletal muscle [27]. According to Tynan et al. [10], the best overall electrohydraulic actuator is a quadrant variant of the HASEL actuator that produces 118% strain, ∼60 N of blocking force, and a power-to-weight ratio of 614 W/kg. In general, electrohydraulic actuators outperform all other types of artificial muscles mechanically, at the cost of relatively low force outputs. 3D printed interdigitated DEAs achieve 9% actuation strain [ϵ] while fully 3D printed silicone DEAs can demonstrate up to 11.11% actuation strain [ϵ] [38,49]. The ferroelectric liquid crystal actuators achieve 19% strain (a 6.33 mm contraction) [25].

#### 5.7.3. Dynamic Response

Fast actuation can increase mechanical and electrical fatigue, accelerating material degradation. With human muscles operating on bandwidths [BW] of tens to several hundred hertz, artificial muscles must also achieve similarly high bandwidth to recreate the rapid reflexes, fine motor control, and dynamic stability of human muscles. HEXEL modules had a bandwidth ([BW], see Table 2) of 15.8 Hz and exhibited problematic “subharmonic actuation behavior” with negative resonance issues with a response at 11 Hz when driven at 22 Hz [45]. Spider-inspired electrohydraulic actuators were built with “roll-off frequencies of up to 24 Hz” and the same subharmonic resonance behavior with a response of 12 Hz when driven at 24 Hz [9]. Increasing the bandwidth ([BW], see Table 2) allows responses across the frequency range and improved errors-corrections from external disturbances, model uncertainty, or desired system change. 3D printed stacked DEAs have been characterized with a frequency range of up to 5 kHz [47]. These stacked DEAs can be good for resonant applications like haptic feedback which require maximal vibration output at a specific high frequency. HEXEL and HASEL systems demonstrate the suitability of electrohydraulic actuators in agile robots meant for jumping, crawling, and rapid reconfiguration. In particular, HEXEL systems have demonstrated a 16 ms actuation rise time [10,45].

As stated earlier, artificial muscles require high bandwidths ([BW], see Table 2) to mimic rapid reflexes and dynamic stability of biological systems. However, maximizing bandwidths introduces significant trade offs. Fast actuation inherently accelerates mechanical and electrical fatigue, leading to premature material degradation and dielectric breakdown [37]. Recent studies on 3D printed polymers materials suggest that material aging significantly alters flexural strength and strain ([ϵ], see Table 2) distribution over time [90]. This indicates high bandwidth ([BW], see Table 2) operation must be balanced against long-term structural integrity of the actuator [90].

Also, defense applications such as UUV propulsion can suffer from parasitic acoustic signatures of high bandwidth ([BW], see Table 2) actuation, undermining requirements for stealthy and quiet operations [44,60,63]. In the case of stacked DEAs, increasing the number of active layers increases force, but introduces a physical ceiling on the frequency and bandwidth.

Electrohydraulics can combine softness with agility due to its relatively high bandwidth. Meanwhile, electrostatics within the ESRA family can achieve <10 ms modulation times for actuator stiffness [31]. On the other hand, Dual-mode electrostatic actuators have been shown to dynamically switch between flexible and stiff states, achieving more than a 2.5 times increase in passive resistance and demonstrating >50% reduction in oscillatory motion due to enhanced damping [31]. Lastly, ESRAs and the broader electrostatic actuator group is positioned in a unique place with its tunable stiffness to help the high dynamic compliance required in wearable biomedical and haptic feedback systems.

### 5.8. Conclusion for Electrostatic Actuation

As summarized in Table 4, electrostatic actuators offer unique advantages such as high bandwidth ([BW], see Table 2), fast response, scalability, and compatibility with soft materials. Compared to other artificial muscles, they are among the most promising candidates for biomimetic artificial muscles in terms of precision, and high-speed applications. The diverse range of electrostatic actuators allow engineers to pick, modify, and optimize the best actuator for any given application, allowing a very wide range of optimized metrics.

However, high voltage requirements create complications in terms of integration and electronics complexity, and there is a tradeoff triangle between force, output strain [ϵ], and bandwidth [BW] (see Table 2). Additionally, electrical limits (such as, dielectric breakdowns, and capacitance limits) and mechanical limits (such as fatigue and viscoelastic creep) continue to be an issue. The majority of works in electrostatic actuators are currently material or device-level with a major leap (or more likely, many small steps) away from system integration due to these various complications. To combat these challenges, future systems may be a hybrid of electrostatic actuators and a non-electrostatic actuator to attempt to create a more capable and flexible actuator, or by being application-specific rather than a generic, universal solution.

**Table 4:** Comparison of electrostatic actuator technologies’ strengths, limitations, and best-fit applications.

Actuator Family	Primary Driving Mechanism	Typical Strengths	Limitations/ Tradeoffs	Best-Fit Applications	Representative Sources
**Dielectric Elastomer Actuators (DEAs)**	Maxwell stress compresses a dielectric elastomer with an applied voltage	Strain [ϵ], fast response, compatible with 3D printing, performance-to-size non-linearly increases when linearly downscaled	Kilovolt-range driving voltage, dielectric breakdown, viscoelastic creep, fatigue, thin-film fabrication	Soft robots, haptics, grippers, underwater propulsion, biomedical devices	[22,32,35,37,38,39,41,43,49,50,56,84,85,87,88,89]
**Ferroelectric & Electrostrictive Polymer** Including PVDF and nematic liquid crystal actuators	Polarization, electrostriction, or piezoelectric/ferroelectric response	Lower voltage, high-frequency potential, can be compact and battery-driven	Material synthesis, thermal stability, long-term reliability, less mature at system-level	Low-voltage muscles, haptics, sensors, miniaturized soft robots	[18,25,27,35,41,73,74,75]
**Liquid Crystal Elastomer (LCE)**	Molecular reorientation or phase transition in anisotropic elastomer networks	Large programmable deformation, shape morphing, fiber bundling, load-to-mass performance	Slower, less mature, alignment, synthesis, electrode interfaces, and scalable fabrication	Morphing skins, soft robotic fibers, adaptive structures, biomedical soft systems	[8,15,18,22,25,33,35,76,77]
**Electrohydraulic** Including HASEL, HALVE, SES, HEXEL	Maxwell pressure drives dielectric fluid inside a soft pouch/shell	Soft, speed, strain [ϵ], self-healing/self-clearing behavior, potential for untethered soft robots	Often voltage-limited, fluid sealing, dielectric breakdown, reliability, and power electronics	Soft grippers, agile robots, reconfigurable modules, musculoskeletal joints	[2,9,10,27,34,45]
**Electrostatic Film Motors & Film Actuators**	Patterned electrode films create electrostatic attraction in stacked film structures	Force-to-weight ratio, low hysteresis, precise, compact geometry, flexible electronics	Precise fabrication and alignment, kilovolt-level drive, stroke/load tradeoff, packaging many thin layers is difficult	Insect-scale robots, endoscopic tools, high-force lightweight actuation, haptic systems, compact robotic mechanisms	[12,30,31,50,74,79,80]
**Stacked Electrostatic Actuators (SEAs)** Including LSEA, stacked dielectric actuators	Many dielectric/electrode layers or plate arrays are stacked to multiply force or displacement	Scalable, contraction ratios, additive manufacturing, macro-scale actuation	Layer alignment, dielectric breakdown, interlayer reliability, capacitance-related bandwidth [BW] limits, fabrication complexity	Power-assist suits, macro-scale soft robots, artificial limbs, high-force electrostatic artificial muscles	[40,42,43,47,91,92]
**Micro & Milliscale Electrostatic Actuators** Including comb drives, FPCB-integrated microactuators, microfluidic microcapacitor arrays	Capacitive force between microfabricated or 3D printed electrodes	Low mass, high integration potential, flexible microsystems, performance-to-size non-linearly increases when linearly downscaled	Small absolute force, demanding fabrication tolerances, difficult wiring, difficult packaging, and scaling to large work outputs	Microrobots, micromirrors, endoscopic tools, conformal electronics, MEMS-scale haptics	[12,23,44,48,50,63,66,72,90]
**Architected Soft Actuators & Hybrid Artificial Muscles**	Mechanical architecture, auxetics, origami, motors, or hybrid material systems convert input motion into extension/contraction	High system-level force and displacement, easier integration with batteries/electronics, strong application demonstrations	Less pure artificial muscle mechanism, may rely on conventional motors or hybrid hardware, added mechanical complexity	Human-scale musculoskeletal robots, prosthetics, exoskeletons, deployable robotic limbs	[33,46,61,63,78]

Although great in research and labs, electrostatic actuators reveal a large bottleneck of manufacturing and scalability. This motivates Section 6 due to electrostatic actuators’ sensitivity to thin-film uniformity, layer alignment, and material interfaces.

## 6. 3D Printed Electrostatic Actuators

Due to a few challenges, manufacturing remains a major barrier to real-world deployment of artificial muscles. First, multilayer fabrication faces print fidelity and manufacturing speed issues in the case of DEAs, IPMCs, and HASEL actuators [33,50]. Second, specialized materials and fabrication methods for microfluidic and SMA actuators have limited availability and repeatability for mass-manufacturing [6]. Third, electrostatic, electrohydraulic, and electrochemical actuators may involve manual assembly processes such as layer alignment and proper encapsulation of dielectric materials, which hinder scalability [51,57]. These fabrication limitations directly impact actuator reliability, scalability, and performance. This section presents a potential solution that directly solves these limitations using 3D printing as a way to get around the manufacturing barrier [50].

### 6.1. Potential Solutions to the Fabrication Challenges

Efforts towards 3D fabrication are limited by the difficulty of removing sacrificial material from narrow embedded channels. Recent research has demonstrated that the use of controlled baking and NaOH flushing has achieved significant clearances in features as small as 200 μm [93]. Neural network-based models are already being proposed to address these issues, as they allow 3D printing optimization to create unique geometries and material combinations to overcome fabrication challenges while reducing material waste and improving structural integrity [78].

Recent 3D and 4D soft robotics involve complex printing methods including FFF, DIW, vat photopolymerization (VPP), material jetting (MJ), and Powder Bed Fusion (PBF) to create different types of soft polymers [33]. Each method differs in build speed, resolution, soft polymer printing material, and support material, which raises the barrier for switching printing methods. The fabrication process is far too complex for layered composites in DEAs, IPMCs, and HASEL actuators [33]. For example, thin-film uniformity of the polymer is critical in DEA manufacturing as non-uniform thickness leads to localized field concentrations and premature electrical field breakdown [41]. Print fidelity is vital in 3D printed DEAs, which expect tight thickness tolerances at the micrometer level; but in reality, produces electrode layer widths off by 40 μm using FFF due to low nozzle resolution [38].

Multilayer fabrication and model accuracy is currently held back by the resolution restriction that is achievable by these 3D and 4D printing techniques. In the case of 3D printed DEAs, 10 μm or less nozzle diameters would greatly increase print precision and performance [38]. Furthermore, specific design methodologies are needed at microscale to overcome issues such as the ‘stair-stepping’ effect on curved surfaces [63]. Systematic characterization of Polyjet technology (a subcategory of MJ) demonstrates that optimizing print orientation is necessary to achieve high accuracy, by moving the jet head parallel to features, such as the microchannel’s longitudinal axis, to produce superior surface morphology and dimensional fidelity compared to perpendicular orientations [92].

**Figure 4:**
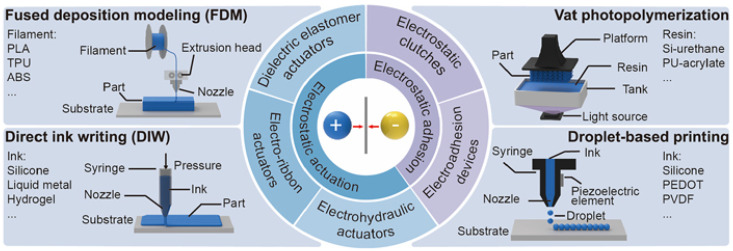
Common fabrication methods for electrostatic actuators. Previously mentioned, FDM is included in place of FFF, while material jetting is included in droplet-based printing. Reprinted from [50].

Another route to help overcome these limitations is architectural advancements, described by Hornik et al. [40], in microfluidic architectures such as the ‘Serpentine’ and ‘Potato spiral’ designs. The Serpentine and Potato spiral have been developed to eliminate sharp turns and bottlenecks, making them significantly more manufacturable with current PolyJet technology compared to earlier double-helix weave designs [40] while also increasing overall potential performance capabilities. An overview of the main manufacturing processes for the creation of electrostatic actuators can be seen in Figure 4.

Multimaterial 3D printing (also known as material doping) allows for actuation to be triggered by different stimuli (temperature, light, magnetic, humidity, electricity, chemical reactions, ultrasound, etc.) based on the composite material [33]. Multimaterial printing enables the production of smart materials that have a big advantage actuating in response to niche environmental conditions. Applications of 3D printed soft robots include grippers, artificial muscles, locomotion robots, biomedical devices, and embedded sensors. Thermally-driven actuators in the field such as SMAs, hydrogels, and LCEs have sparsely employed 3D smart materials for functional applications. Instead, 3D printed smart materials have been mainly seen in simple demonstrations like contracting artificial muscles [33].

A novel approach to fabricate electrostatic microactuators takes advantage of FFF, two-photon polymerization (TPP), metal sputtering, and FPCBs [23]. Under this process, flexible microsystems are produced by 3D printing electrostatic microactuators directly upon the FPCB substrate. This work overcomes the challenges of aligning and incorporating rigid microactuators along compliant electronics to create flexible, integrated micromirror arrays and legged microrobots that weigh only 4 mg. Notably, the crawling microrobot achieved locomotion speeds of 0.27 body lengths per second, while the rotary micro-mirror actuators maintained ≤20% angular displacement loss despite an applied substrate curvature [23,66]. The results highlight FPCBs as a highly customizable, mass-produced option as the backbone for actuated microsystems that can also integrate seamlessly with commercial off-the-shelf electronics through standard soldering methods. Combining fabrication processes brings about additional manual labor and its own set of new challenges, but utilizing multi-printing fabrication and post-processing techniques may be the best approach forward to leverage different fabrication advantages.

Reviews of 3D printing for electrostatic devices and EAPs note limited commercial dielectrics and conductive inks/filaments/resins with the right combination of permittivity, breakdown strength, mechanical robustness, and printability [49,50]. While also noting the already established challenge of reliably fabricating thin, uniform, multilayer, yet monolithic structures with good interfacial bonding [24,43,93]. Introducing new 3D printing technology and material types designed for artificial muscles would simplify fabrication and research processes. New technologies that would help emphasize this would be compatibility with soft materials, multi-material printing, micro-scale printing, and accurate thin-film fabrication. While new material types would emphasize flexibility, softness, and conductivity.

### 6.2. Scaling and Stacking of Electrostatic Actuators in Regards to 3D Printing

#### 6.2.1. Stacked Dielectric Actuators

At a smaller scale, monolithic 3D printed SDEAs (introduced in Section 5.5) can use multi-material extrusion of conductive and dielectric Thermoplastic polyurethane (TPU) [94] to create multi-layer capacitive stacks in a single print. This method reduces assembly effort and allows complex electrode routing. Combined electrical–mechanical lumped-element model captures bandwidth ([BW], see Table 2), blocked force, and free stroke as functions of layer count, dielectric thickness, and stiffness anisotropy. Printed devices have demonstrated operation up to kilohertz frequencies. These designs highlight how stacking not only increases force, but also provides a controlled platform to study electromechanical coupling [47].

Reviews on additive manufacturing of electrostatic devices [22,35,50] further emphasize that resolution, ink rheology, and material availability are key bottlenecks for miniaturized EAP actuators, but demonstrate that FFF [43,50], VPP [50], MJ, and DIW [38,49] are already being applied to DEAs, HASELs, and interdigitated structures with increasing success.

#### 6.2.2. Stacked Elastomer Actuator and Large-Scale Stacked-Type Electrostatic Actuator

It has been demonstrated that monolithic 3D printing can be achieved through FFF [43] for SDEAs an LSEAs. Using this method, stacked actuators were able to be produced in a single process, allowing parts to be scaled, individualized, and combined into larger structures without changing the underlying fabrication technology, facilitating the embedding of active components directly into their prints [43]. Traditional electrostatic actuators often struggle to generate high stroke actuation and high force density simultaneously, as the gap between electrodes can expand excessively [42]. To solve this issue, advanced SEAs/LSEAs utilize thin bendable hinges to connect rigid electrode plates that allow the actuator to contract easily while preventing excessive extension of the electrodes during loading [42].

Even with the benefits provided by the 3D printing of SEAs/LSEAs, interlayer reliability is an inherent issue through this process, as a compromised dielectric layer is sufficient to cause electromechanical instability or dielectric breakdown for the entire stack [43]. As actuators scale in size and layer count, a large limiting factor becomes the electrical cutoff frequency, the maximum operating frequency of the actuator, which is dictated by the active area and number of active layers [47]. Lastly, environmental and viscous effects affect the actuator as hysteresis is present in larger stacks due to air viscosity, as air must flow into and out of the gaps between the electrodes during high-speed actuation [42].

### 6.3. Micro-Milliscale Electrostatic Actuators

Advancements in 3D printing have enabled fabrication at a smaller scale, leading towards the idea of electrostatic micro-actuation. A major foundation architecture for electrostatic micro-actuation is the comb drive. These comb drives utilize sets of interdigitated finger electrodes to convert electrical potential into linear motion [63]. By increasing the number of interdigitated fingers, high displacements and forces can be achieved through cumulative capacitive effects at microscale [63]. Traditional comb drives are often silicon based MEMS components, but their principles are being increasingly extended to 3D printed soft actuators and dielectric elastomers to enable complex, biddable motions in flexible microsystems [38].

The research shows several efforts to push electrostatic actuation into the micro- and millimeter scales for robotics, optics, and wearables, where low mass and planar geometry are critical. One major thread [23] uses TPP 3D printing directly on FPCBs, followed by metal sputtering to form 3D electrostatic rotary microactuators and micromirror arrays that remain functional while the FPCB substrate is bent, making it possible to embed these devices in conformal electronics or soft surfaces [23].

This integration has enabled quadruped micro-robots with total mass of only a few milligrams that achieve significant fractions of a body length per second in locomotion, as well as flexible optical arrays for beam steering [23]. This illustrates that electrostatic micro-actuators can be embodied in mechanically biddable, solderable systems suitable for haptics and conformal devices rather than only rigid chips [12]. At millimeter scale, the insect-muscle-inspired stacked electrostatic film actuators mentioned above represent a bridge between MEMS and macro soft robots, achieving power densities ([$P_{d}$], see Table 2) on the order of tens of watts per kilogram with low-profile, flexible laminates integrated into crawling and endoscopic systems [66].

### 6.4. Manufacturing with 3D Printing

Easing the manufacturing process of these electrostatic systems for more testing and data collection within real-world applications would be an important effort. Research in this area is promising, as one such approach involves converting 3D printed embedded microfluidic channels into electrical wiring through iterative deposition of conductive carbon nanofibers (CNF) in organic solvents [95]. These solvents can be evaporated to deposit the CNF to enable 3D printed actuators, flexible electronics, and, in general, devices requiring distributed embedded electrical input/output [95].

Single-process manufacturing is much more accessible and requires less effort with up to 150 layers of DEAs that can be 3D printed without manual assembly [47]. However, it is worth exploring multi-technique fabrication to take advantage of key fabrication advantages that would increase the success of application-based artificial muscles. TPP 3D printing in combination with made-to-order FPCBs manufacturing is an intriguing combination that creates accessible, MEMs-scale electrostatic actuators [23].

Extending the fabrication methods of TPP, along with the principles of comb drives to 3D printed soft actuators and dielectric elastomers would enable actuator designs that are well-suited for micro-scale applications, physical flexibility in real-world environments, and allow for easy integration with commercial electronics for fully embedded systems. Multi-material printing is a worthwhile method of producing composite materials that respond to multiple stimuli, which can improve performance across a wide variety of real-world environments. As the manufacturing process is optimized across all types of artificial muscles for size, weight, and power constraints; then the fabrication of scalable, low-cost, and reproducible artificial muscles will be more readily available.

### 6.5. Material Science and Machine Learning

The reduction of burden in experimental material science research is paramount to make breakthroughs in artificial muscles. A data-driven research approach that utilizes artifical intelligence (AI) and machine learning (ML) has been proposed by Huang, et al. to accelerate the discoveries of artificial muscle materials [32]. It was discussed that by leveraging material databases and ML models to map descriptor-target relationships, researchers can predict target performance metrics, material composition, structures, and fabrication strategies for improved dielectrics, synthetic composite nanomaterials in IPMCs, and polymer-based composite materials in DEAs [32]. Particularly noted, ML models trained on continually updated experimental data along with standardized materials databases can help identify promising polymer-based composite materials for DEAs before physical prototyping [32]. Researchers believe that the potential of low-cost and accessible ML tools is poised to become integral to artificial muscle research-supporting predictions of continuous performance variables, discrete design choices, and fabrication pathways [22,32].

Beyond predicting materials, many point out that AI-driven techniques offer the potential to extract insights across the literature via natural language processing [96], uncover generalized performance trends without exhaustive experimentation [22], and enable smart materials with embedded sensing and feedback [35]. Their collective conclusion implies that data-driven and AI-enabled research is positioned to act as a catalyst for the next major breakthroughs in artificial muscle technologies, transforming how these systems are designed, optimized, and deployed. This shift toward human-machine collaboration is demonstrated with Rojek et al. using AI-based generative design and digital twins to refine ergonomics and material performance in real time in the optimization of 3D printed hand exoskeletons [78].

Furthermore, Jeong et al. states that ML models could potentially be used to facilitate traditional computation methods and estimate optimal parameters for mechanical-property-modeling of DEAs [37]. This research has been conducted to capture the long-term, dynamic behavior of DEAs due to its viscoelastic nature by combining non-linear and time-dependent energy-based theories. Its results suggest that up to 20 minutes of dynamic DEA response can be accurately predicted using a real-time ML estimation method that targets physical similarity rather than complex, real-world physics models to reduce computation time [37]. The team also attempted to optimize parameters for mechanical-property models of the dynamic DEAs using highly-accurate deep learning, but moved away from that method due to excessive computation times. As AI and ML evolves in computational efficiency, accessibility, and accuracy, it would be worth considering adding its strengths and advantages towards research in artificial muscles to assist in material science discovery and enhanced physics modeling [32,37].

### 6.6. 3D Fabrication in Applications

To move beyond laboratory prototypes into real-world applications, future work should emphasize scalable manufacturing techniques for microscale to macroscale actuators. While actuator quality and efficiency ([η], see Table 2) are heavily dependent on the fabrication process, ensuring repeatability across the manufacturing process will be integral. Palmić, T. B., and Slavič, J. improves the manufacturing repeatability of 3D printed, stacked, dielectric actuators by using a single-process FFF accompanied by custom G-code generation and nozzle ironing techniques to improve layer consistency [47]. While these techniques are specific to FFF, they also propose inspection systems for visual feedback that can provide “live, quantitative indicators of the quality of the deposited layers”, which can be translated to other fabrication techniques [47]. This is an avenue that will likely be aided with new developments in AI and ML [50].

On the milliscale, electrostatic film actuators can be created from careful hierarchical layering of milliscale electrodes and electrode arrays to behave like natural insect muscles [12]. Using printed circuit MEMS techniques to create copper-etched films, the produced artificial muscles are capable of 85 mN force and 15 mm stroke from a 92 mg mass, making it well suited for endoscopic surgical tools [12]. The hierarchical layered approach assists in scalability to further replicate larger insect and potentially human muscles.

On the mesoscale, cable-driven DEA mechanisms employ a modular approach to separate the actuator from the end-effector for design flexibility [24]. The increased flexibility allows the artificial muscle to swap into a “pinching gripper, a multi-degree-of-freedom mechanism, and a soft end-effector” to pick up diverse real-world objects like an earbud, a thumb tack, and a pencil [24].

On the macroscale, architected soft actuators can assemble multiple actuator units with an onboard battery to replicate a human-scale leg capable of kicking a ball using 3D printed handed shearing auxetic (HSA) and origami bellows structure [46]. At this scale, having modularity and reconfigurability is especially important to maintain scalability across applications. To connect actuators together, macro-level structures for artificial muscles could take inspiration from the human musculoskeletal system to form bone-like, rigid links and tendon-inspired connectors through 3D printed parts [46]. HEXEL modules employ a unique approach with magnetic snap-on assembly for rapid, reconfigurable robot systems for contraction, lateral expansion, and rolling [45].

Manufacturing and scalability should be treated as a priority design parameter for successful application-driven results.

## 7. Conclusions

Advantages and disadvantages of various non-electrostatic actuators and electrostatic actuators were discussed and compared. While advancements in manufacturing, particularly 3D printing, were shown to have a promising impact in this field.

### 7.1. Authors’ Perspective

We believe that the engineering of high-performance artificial muscles will require coordinated advances in material science, physics modeling, manufacturing innovation, and benchmarking standards. The increasing demand for actuators capable of organic deformation has propelled research into muscle-mimetic technologies for modern applications such as marine propulsion, aerospace morphing, and medical prosthetics.

Another advancement we feel is required for promoting the development of high-performance actuators includes having a larger research focus towards a system-level integration of artificial muscles such as; feedback control, embedded system, self-sensing capabilities, real-time control, co-designed mechanism, compactness, modularity, and packaging for environmental protection. Especially when considering that actuators do not have a universal solution for each application, it becomes vital that we have a strong foundational understanding of the systems integration.

An actuator should be chosen for each application based on the metrics that need to be optimized (Table 2). Some actuators would have a greater desired metric for an application than another, while also having metrics that are not as important that can be ignored in favor of other metrics.

Although each actuator family has its advantages and disadvantages in terms of metrics (Table 2), they can still be tailored by combining different systems of various activation mechanisms and fabrication methods to better optimize the desired metrics of an actuator for each given application. This results in a hybrid of actuators that would most likely utilize the advantages of an electrostatic actuator with a non-electrostatic actuator.

We think that an advancement for the full deployment of artificial muscles is its manufacturability and modularity. As discussed in Section 6, manufacturing electrostatic actuators is correlated with and influenced by advances towards 3D printing. Therefore, by dedicating a few 3D printer’s design criterion to be optimized for electrostatic actuators through prioritizing micro-scale, multi-material, increasing materials specialized for electrostatic actuators, and ease of complex material printing, the field of experimental research of artificial muscles may accelerate rapidly while also taking the first steps towards manufacturability.

Similarly, designing a system that supports the use of modular actuators would greatly reduce the need to manufacture many different types of components in a system and instead can focus on a select few that can be utilized in many different designs. We think this is particularly important for artificial muscle applications given its current state of complexity and difficulty to fabricate.

### 7.2. Summary

An assessment of each actuator family in the standardized metrics (defined in Table 2) is presented in Table 5. Because actuator families differ substantially in scale, operating principle, and intended application, the rankings in Table 5 are qualitative and relative, rather than absolute. Each column of metrics follows a forced relative comparative ranking, assigning 4 High, 4 Neutral, and 4 Low ratings to identify the strongest, intermediate, and weakest actuator for that specific metric. These rankings are based on representative literature values and mechanism-level tradeoffs. Soft approximate quantitative values are provided to guide the qualitative rankings in Table 5.

**Table 5:** Forced relative ranking of each standardized metric for each actuator family. Each family is given either a “high”, “neutral”, or “low” performance rating for each metric. Each metric is defined as follows: “**High**” excels in this metric, “**Neutral**” neither excels nor falls short in this metric, and “**Low**” falls short in this metric.

	Standardized Metrics	Output Strain (ϵ)	Output Stress (σ)	Stress- Strain Relation- Ship (*k*)	Force-to- Weight Ratio (FWR)	Power Density ($P_{d}$)	Efficiency (η)	Bandwidth (BW)
Actuator Families								
**Ionic & Electrochemical**		Low	Low	Low	Low	Low	Neutral	Low
**Pneumatic Artificial** **Muscles (PAMs)**		Neutral	High	Neutral	Neutral	Neutral	Low	Low
**Hydraulic &** **Fluid-Driven**		High	High	High	Neutral	High	Neutral	Low
**Thermally Driven**		Neutral	High	Low	High	High	Low	Low
**Dielectric Elastomer** **Actuators (DEAs)**		Neutral	Neutral	Neutral	Neutral	Neutral	High	High
**Ferroelectric &** **Electrostrictive Polymer**		Low	Low	Low	Low	Low	High	High
**Liquid Crystal** **Elastomer (LCE)**		High	Low	Neutral	High	Neutral	Neutral	Neutral
**Electrohydraulic**		High	Neutral	High	High	High	High	Neutral
**Electrostatic Film** **Motors & Film Actuators**		Low	High	High	High	Neutral	High	Neutral
**Stacked Electrostatic** **Actuators (SEAs)**		Neutral	Neutral	High	Neutral	Low	Low	High
**Micro and Milliscale** **Electrostatic Actuators**		Low	Low	Low	Low	Low	Neutral	High
**Architected Soft** **Actuators & Hybrid** **Artificial Muscles**		High	Neutral	Neutral	Low	High	Low	Neutral

Note. The high, neutral, and low performance ratings are qualitative relative rankings informed by soft quantitative ranges rather than strict thresholds. Approximate guide ranges were: **output strain**, low <20%, neutral 20–30%, high >30%; **output stress**, low <10 kPa, neutral 10–100 kPa, high >100 kPa; **force-to-weight ratio**, low <10, neutral 10–100, high >100; **power density/power-to-weight ratios**, low <50 W/kg, neutral 50–200 W/kg, high >200 W/kg; **efficiency**, low <25%, neutral 25–60%, high >60%; and **bandwidth**, low <10 Hz, neutral 10–100 Hz, high >100 Hz. These ranges were used as guidance within a forced relative ranking system rather than as absolute exclusion criteria. The system’s purpose is to provide numerical references toward the performance ratings and to aid interpretation.

### 7.3. Concluding Remarks

This work recognizes the conflicting nature of metric evaluation across inherently different actuator mechanisms, and conducts a mixed quantitative-qualitative analysis that allows actuators to be mapped directly to real-world applications while using relevant design criteria. Progress towards the widely-adopted artificial muscle will not depend on only isolated breakthroughs, but on the convergence of data-driven discovery, physics-informed modeling, system-level integration, and scalable manufacturing. Machine learning (ML) can support material designs with hybrid actuation, embedded sensing and control, and application-driven fabrication strategies that will allow artificial muscles to move beyond laboratory demonstrations and achieve robust, safe, and biologically comparable performance in real-world systems. Lastly, increasing the current 3% focus on system-level integration (Table 1) will be critical for the widespread adoption in soft robotics, wearables, and biomedical technologies.

## Data Availability

The original contributions presented in this study are included in the article. Further inquiries can be directed to the corresponding authors.

## Abbreviations

The following abbreviations are used in this manuscript:

AI	Artificial intelligence
*BW*	Bandwidth
BIRAN	Bidirectional rotational Antagonistic
CNF	Carbon nanofibers
DC	Direct current
DEA	Dielectric elastomer actuator
DIW	Direct ink writing
DOF	Degree of freedom
EAP	Electroactive polymer
EPAM	Electroactive polymer artificial muscle
ERA	Electro-ribbon actuator
ESRA	Electro-stiffened ribbon actuator
FFF	Fused filament fabrication
FDM	Fused deposition modeling
FPCB	Flexible printed circuit board
*FWR*	Force-to-weight ratio
HALVE	Hydraulically amplified low-voltage electrostatic
HASEL	Hydraulically amplified self-healing electrostatic actuator
HEEI	High-energy electron-irradiated
HEXEL	Hexagonal electrohydraulic
HSA	Handed shearing auxetic
IEC	International Electrotechnical Commission
IPMC	Ionic polymer-metal composite
LCE	Liquid crystal elastomer
LSEA	Large-scale stacked-type electrostatic actuator
MAV	Micro-air Vehicles
MEMS	Micro-electro-mechanical-systems
ML	Machine learning
PAM	Pneumatic artificial muscle
PBF	Powder Bed Fusion
PVDF	Polyvinylidene fluoride
PVDF-TrFE	Polyvinylidene fluoride-trifluoroethylene
ROV	Remotely Operated Vehicle
SEA	Stacked elastomer actuator
SMA	Shape memory alloy
SMP	Shape memory polymer
TPP	Two photon polymerization
TPU	Thermoplastic polyurethane
UL	Underwriters Laboratories
UUV	Unmanned underwater vehicles
VPP	Vat photopolymerization
3D	Three dimensional
4D	Four dimensional
